# Genome editing of *CCR5* by CRISPR-Cas9 in Mauritian cynomolgus macaque embryos

**DOI:** 10.1038/s41598-020-75295-z

**Published:** 2020-10-28

**Authors:** Jenna Kropp Schmidt, Nick Strelchenko, Mi Ae Park, Yun Hee Kim, Katherine D. Mean, Michele L. Schotzko, Hyun Jun Kang, Thaddeus G. Golos, Igor I. Slukvin

**Affiliations:** 1 Wisconsin National Primate Research Center, University of Wisconsin-Madison, Madison, WI USA; 2 Department of Comparative Biosciences, Wisconsin National Primate Research Center, University of Wisconsin-Madison, 1220 Capitol Court, Madison, WI 53715 USA; 3 Department of Obstetrics and Gynecology, University of Wisconsin-Madison, Madison, WI USA; 4 Department of Pathology and Laboratory Medicine, Wisconsin National Primate Research Center, University of Wisconsin-Madison, 1220 Capitol Court, Madison, WI 53715 USA; 5 Department of Cell and Regenerative Biology, University of Wisconsin-Madison, Madison, WI USA

**Keywords:** Embryology, HIV infections

## Abstract

The discovery that CCR5 serves as an R5-HIV-1 co-receptor, coupled with findings of protection from HIV infection in individuals lacking CCR5, led to the exploration of novel therapeutic strategies for HIV infection based on genome editing of *CCR5*. Advancing translation of *CCR5*-mutant-based cellular therapies for HIV requires development of novel physiologically relevant animal models. Mauritian cynomolgus macaques (MCMs), with high degree of MHC allele sharing, are valuable models for HIV-1 research and stem cell therapies. To facilitate the generation of a CCR5-mutant MHC-defined MCM model, we explored editing the *CCR5* gene in MCM embryos via CRISPR-Cas9. We refined ovarian stimulation and in vitro fertilization (IVF) methods established for Chinese cynomolgus macaques to generate in vitro MCM embryos. Time-lapse embryo imaging was performed to assess the timing of MCM embryonic developmental events in control and CRISPR-Cas9 microinjected embryos. Using a dual-guide gene targeting approach, biallelic deletions in the *CCR5* gene were introduced into ~ 23–37% of MCM embryos. In addition, single blastomere PCR analysis revealed mosaicism in CCR5 editing within the same embryo. Successful development of IVF and *CCR5* editing protocols in MCM embryos lays a foundation for the creation of *CCR5*-mutant MCMs to assess novel stem cell-based HIV therapeutics.

## Introduction

Resistance to human immunodeficiency virus-1 (HIV-1) infection in humans has been associated with a homozygous 32 base-pair deletion in the chemokine (C–C) motif receptor 5 (*CCR5*) gene^1–3^. CCR5 is a co-receptor for macrophage- and dual-tropic HIV-1 isolates^4,5^. Upon binding of the HIV-1 viral envelope protein to the CD4 receptor on the cell surface, the activation of the CCR5 co-receptor facilitates viral fusion to the cell plasma membrane allowing for entry of viral contents into the cell. The homozygous 32 base-pair deletion leads to a truncation of the CCR5 protein and ultimately the loss of expression on the cell’s surface^6^. This deletion is reported to occur in < 1% of the population^1,2^ and in those individuals, HIV-1 resistance has been observed despite multiple instances of viral exposure^1^. Hence, *CCR5* has become an attractive candidate for assessing mechanisms of HIV-1 infection and also for developing drug treatments and gene-based therapies^6–9^.

The cure of HIV by transplanting *CCR5*-mutant (*CCR5*-delta32) hematopoietic stem cells (HSCs)^10,11^ demonstrated the feasibility and power of stem cell-based therapies for eliminating latent virus and controlling AIDS. However, to broaden and refine the application of this therapeutic approach in HIV-infected patients, it will be critical to define the spectrum of anti-viral protection, the engraftment threshold of CCR5-mutant HSCs required for protection, and the potential for depletion of the virus reservoir through “allo-effect” following allogeneic hematopoietic stem cell (HSC) transplantation using a physiologically relevant animal model.

Nonhuman primates, and more specifically macaques, serve as important model species to study HIV-1 through infection with simian immunodeficiency virus (SIV)^12^. SIV infected animals show similar elements of human HIV-1 infection including immune responses and pathogenesis, and additionally, tissues are more accessible for study compared to human studies^12^. Mauritian cynomolgus macaques (MCMs) offer a distinct advantage over other macaque species as they have only seven major histocompatibility complex (MHC) haplotypes allowing for the study of defined immune responses^12,13^ and to control genetic factors in the setting of allogeneic bone marrow transplant. This provides a powerful means for quantifying the effect of MHC matching on the capacity of allogeneic cells to purge the SIV reservoir.

Genome editing by way of CRISPR-Cas9 technology has proven successful for introducing gene disruptions in nonhuman primates (NHP) for the study of mutated gene function and the development of models of human diseases^14^. Microinjection of CRISPR-Cas9 genome editing constructs into one-cell macaque embryos can be performed to introduce genetic mutations in target genes associated with human disease, where the edited offspring can be evaluated for phenotypic changes associated with mutated gene function and/or used as a source of mutated cells for transplantation studies^15–19^. We have previously demonstrated targeting of *CCR5* in human induced pluripotent stem cells (iPSCs), where macrophages derived from *CCR5*-edited iPSCs were resistant to viral challenge^20^. In the current study, to create a platform for generating *CCR5*-edited MHC defined NHP model, we established in vitro fertilization (IVF) procedures in MCMs, including ovarian stimulation and embryo culture protocols, along with procedures for the efficient editing of the *CCR5* locus in MCM embryos.

## Results

### Ovarian stimulation of MCM oocyte donors

**Figure 1:**
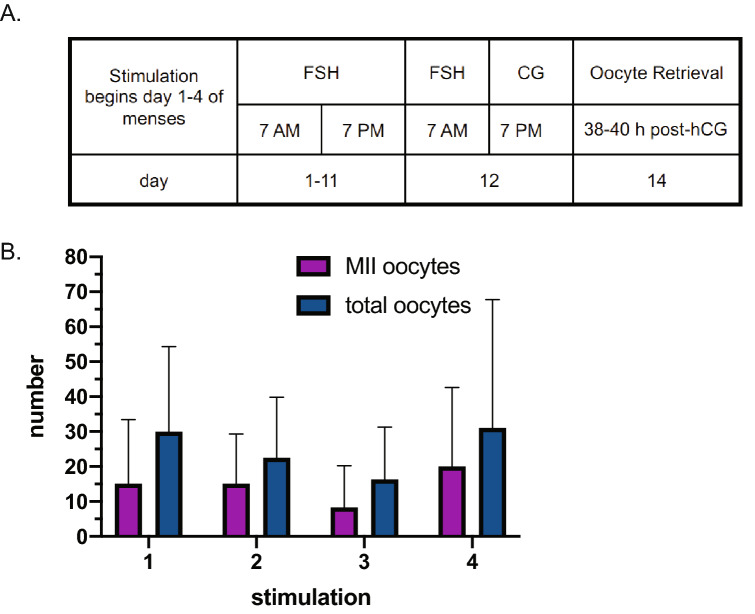
Ovarian stimulation of MCM oocyte donors. (**A**) Schematic of the ovarian stimulation regimen. (**B**) Number of mature MII and total MCM oocytes recovered for each stimulation. Animals received up to four rounds of ovarian stimulation.

The methods for producing IVF embryos from Chinese cynomolgus macaques (CCMs) are well established, whereas there are no publications to date that specifically describe in vitro generation of MCM embryos^21,22^. Applying an ovarian stimulation protocol for CCMs to MCM oocyte donors resulted in recovery of relatively few to no mature oocytes upon laparoscopic follicular aspiration. Twice daily treatment with recombinant human follicle stimulating hormone (FSH) for 7–9 days and performing the oocyte retrieval at 36–41 h post-recombinant human chorionic gonadotropin (hCG) treatment resulted in a mean recovery of 13.4 (± 8.2 SD) oocytes of which 4.2 (± 6.1 SD) were mature, metaphase II (MII) oocytes (n=5 ovarian stimulations). Extending the FSH treatment from 8–10 to 11–12 days and performing the oocyte retrieval between 38–40 h post-hCG treatment resulted in the collection of a greater proportion of mature MCM oocytes (Fig. 1A). Using the ovarian stimulation protocol tailored to MCMs, a mean of 24.3 (± 20.7 SD) MCM oocytes were recovered from the follicular aspirate with approximately 56% of the total oocytes being mature MII oocytes (Fig. 1B). MCM oocyte donors could undergo up to four stimulations, however, there were no significant differences in the total oocytes or number of mature MII oocytes recovered between the first to fourth ovarian  stimulation events (Supplemental Table S1).

### CRISPR-Cas9 delivery and in vitro development of MCM embryos

**Figure 2:**
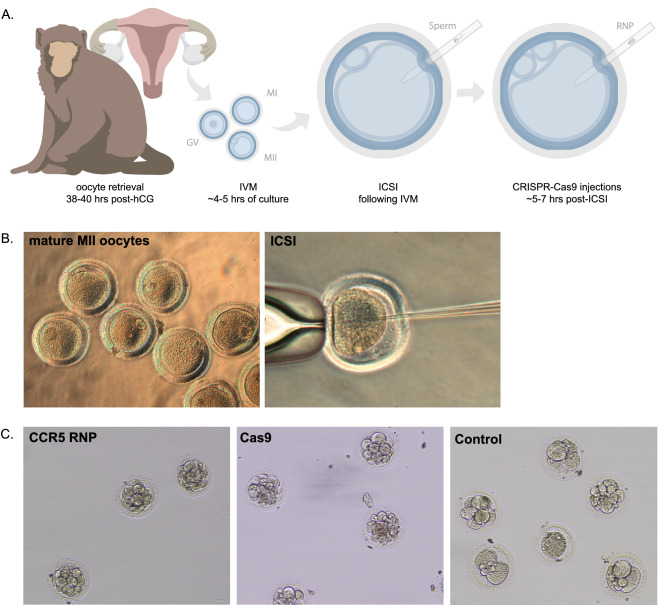
In vitro production and CRISPR-Cas9 microinjections of MCM embryos. (**A**) Timeline of oocyte retrieval and early embryo manipulations. Oocytes were retrieved laparoscopically between 38 and 40 h post-treatment of hCG and then cultured for ~ 4–5 h to allow for in vitro maturation (IVM) of oocytes. Following IVM, mature oocytes were fertilized by ICSI and incubated for ~ 5–7 h prior to embryo microinjection with CRISPR-Cas9 constructs (Cas9 alone or CCR5 RNP), or alternatively, embryos were cultured as control. Representative images of (**B**) MII MCM oocytes and (**C**) embryonic development of *CCR5* RNP, Cas9 alone and control embryos.

After ~ 4–5 h of in vitro maturation (IVM), mature oocytes were obtained from both CCMs and MCMs that were fertilized by intracytoplasmic sperm injection (ICSI). At ~ 5–7 h post-ICSI, embryos were either microinjected with Cas9 protein alone (no sgRNA), microinjected with a ribonucleoprotein (RNP) mixture comprised of Cas9 protein and dual-guide RNAs designed to target the *CCR5* gene, or cultured as control (Fig. 2A). Representative images of mature MII oocytes that underwent fertilization by ICSI are shown in Fig. 2B,C. A total of 240 MCM oocytes were fertilized by ICSI, where 72.7% of control, 49.2% of Cas9 alone microinjected and 45.8% of *CCR5* RNP microinjected oocytes cleaved (Table 1). In comparison, a total of 162 CCM oocytes were fertilized by ICSI of which 78.4% of control, 52.5% of Cas9 alone microinjected and 37.6% of *CCR5* RNP microinjected cleaved (Table 1). Cleavage rates were significantly reduced in MCM and CCM *CCR5* RNP injected oocytes compared to control oocytes with a trend towards a significant reduction (p = 0.053) in Cas9 alone MCM oocytes compared to control MCM oocytes (Table 1). A subset of early cleavage stage embryos were transferred to recipient females, hence the blastocyst rate was only calculated in experiments where embryos were not removed for transfer and were instead monitored in terms of their development in vitro. Representative images of cleavage stage embryos for each experimental group are shown in Fig. 2C. Embryos were collected for molecular analysis of CRISPR-Cas9 editing when developmental arrest was observed or upon blastocyst hatching. The blastocyst rate for control MCM and CCM embryo development was 19.8% and 0%, respectively (Table 1). Of note, the CCMs underwent the same extended ovarian stimulation regimen that was optimized for MCMs and the CCM oocytes were fertilized with MCM sperm. The reduced development rates in CCMs may be attributed to the extended ovarian stimulation regimen tailored to MCMs that could negatively impact oocyte age and quality. The ovarian stimulation protocols described for CCMs by others^21,22^ were not used in this study to have consistent treatment of CCMs and MCMs. While the blastocyst formation rate tended to be lower in either Cas9 alone or *CCR5* RNP microinjected MCM embryos compared to control MCM embryos, there were no statistically significant differences.

**Table 1:** Summary of in vitro development of cynomolgus macaque embryos.

	Total from all experiments				Total from experiments with no embryo transfer				
	Oocytes	Cleaved	Cleavage rate (%)	Blastocysts	Oocytes	Cleaved	Cleavage rate (%)	Blastocysts	Blastocyst rate (%)
**MCM**									
Control	44	32	72.7^a^	16	19	16	84.21	3	19.8
Cas9	65	32	49.2	0	40	13	32.5	0	0
*CCR5* RNP	131	60	45.8^a^	3	78	15	19.2	1	6.7
**CCM**									
Control	37	29	78.4^b^	0	22	7	31.8	0	0
Cas9	40	21	52.5	2	19	3	15.8	2	67
*CCR5* RNP	85	32	37.6^b^	0	2	2	11.76	0	0

The total number of oocytes fertilized by ICSI, cleaved embryos or blastocyst stage embryos are represented in the portion of the table without shading; since some embryos were removed for embryo transfer it is not possible to determine an accurate blastocyst rate. A pair-wise analysis using Fisher’s Exact Test was performed for each comparison within MCM or CCM experimental groups and a Bonferroni correction for multiple comparisons was applied to the p-value. A similar superscript denotes significance between the comparison: ^a^p = 0.008, ^b^p = 0.00018.

*MCM* Mauritian cynomolgus macaque, *CCM *Chinese cynomolgus macaque, *RNP *CRISPR-Cas9 ribonucleoprotein complex.

**Figure 3:**
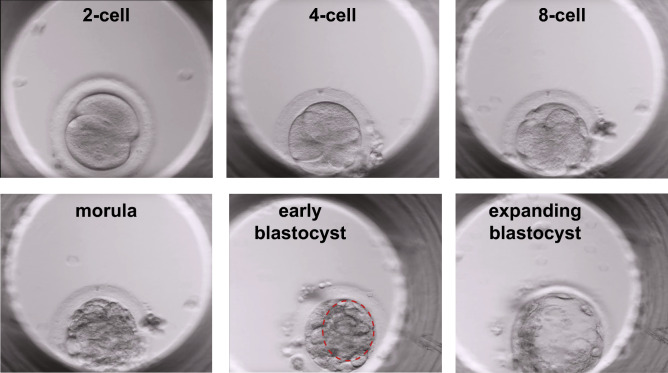
Time-lapse embryo imaging of MCM embryonic development. Representative images of 2-cell, 4-cell, 8-cell, morula, initial blastocyst (red dashed circle outlines the forming blastocoel cavity) and expanding blastocyst stages.

Time-lapse embryo imaging was performed to assess the timing of developmental events in control and microinjected embryos. Control, unmanipulated embryos reached the 2-cell, 4-cell, 8-cell, morula and blastocyst stages at the following hours post-insemination, respectively: 33.6 h ± 12.1 SD, 47.7 h ± 29.7 SD, 58.0 h ± 20.5 SD, 106.0 h ± 26.9 SD, and 216.5 h ± 47.4 SD (Table 2). Representative morphology at each developmental stage is shown in Fig. 3. The development of Cas9 alone and *CCR5* RNP-microinjected embryos tended to be delayed in comparison to control embryos. The difference in timing between control and *CCR5* RNP injected embryos approached significance for the timing of cleavage to 2-cells and development to 8-cells (p = 0.070 and p = 0.054, respectively), but were not statistically significantly different.

**Table 2:** Timing of embryonic developmental events in cynomolgus macaque embryos cultured in vitro.

	2-cell h ± SD (n)	4-cell h ± SD (n)	8-cell h ± SD (n)	Morula h ± SD (n)	Blastocyst h ± SD (n)
Control	33.6 ± 12.1 (12)	47.7 ± 29.7 (9)	58.0 ± 20.5 (9)	106.0 ± 26.9 (6)	216.5 ± 47.4 (2)
Cas9	45.9 ± 19.6 (5)	50.9 ± 4.6 (8)	68.8 ± 35.9 (12)	110.0 ± 35.3 (6)	–
*CCR5* RNP	56.6 ± 20.4 (15)	64.5 ± 22.0 (22)	81.7 ± 22.4 (19)	116.9 ± 28.9 (15)	177.0 (1)

The time for each cleavage or developmental event is represented in hours, where the time of ICSI is time 0 h. The mean time ± SD is represented with the number of embryos analyzed in parenthesis. A two-way ANOVA resulted in no significant differences between embryo groups or developmental time points. The time to the blastocyst stage was not statistically evaluated given the limited sample number.

### Genome editing analysis of CCR5 in whole embryos and individual blastomeres

**Figure 4:**
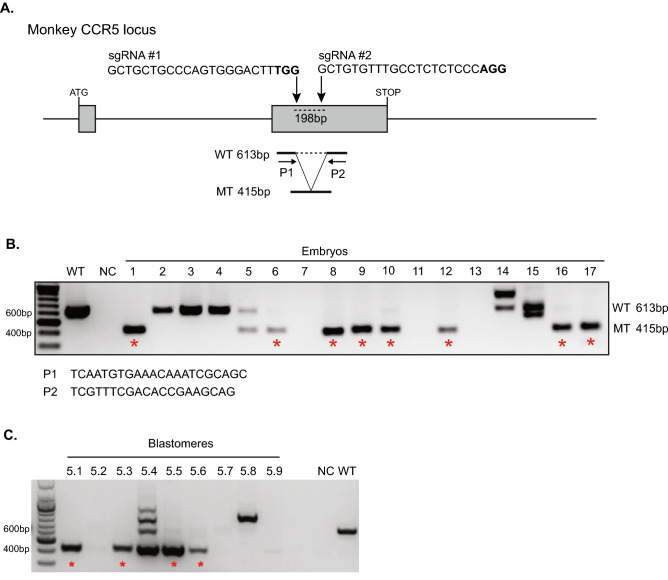
Genotyping of CCR5-editing in whole MCM embryos. (**A**) Schematic of CRISPR-Cas9 targeting design. Representative gel electrophoresis images of the CCR5 amplicon in (**B**) whole embryos and (**C**) individual blastomeres of a single embryo. The wild-type (WT) product is 613 bp, whereas the 198 bp deletion results in a 415 bp product. An asterisk denote a homozygote. *P1* primer 1, *P2* primer 2, *MT *mutation, *NC *negative control, *WT *wild-type positive control. A 100 bp ladder was used.

To disrupt the *CCR5* gene, we used two gRNAs to target sequences within exon 2, including a 24 base-pair (bp) deletion region that was found to prevent functional *CCR5* expression in NHPs^23^ (Fig. 4A). Genome editing at the *CCR5* locus was evaluated by PCR and gel electrophoresis to determine if editing occurred with an expected excision of a 198 bp region between the two *CCR5* target sites. A schematic of the *CCR5* target sites and expected product sizes is shown Fig. 4A. A 613 bp PCR amplification product is expected for wild-type *CCR5* alleles, whereas a 415 bp product indicates editing of the 198 bp deletion. The presence of both 613 bp and 415 bp products suggests incomplete editing in the embryo and the creation of a heterozygote, and the presence of only the 415 bp product indicates biallelic (homozygous) editing. Both whole embryos and individual blastomeres were analyzed by PCR and gel electrophoresis with representative gel images displayed in Fig. 4B,C, respectively (original gel images are displayed in Supplementary Fig. S1). A total of 73 whole embryos from nine oocyte donors were analyzed. A *CCR5* PCR amplification signal was detectable in 60 embryos, of which 53.3% contained the predicted 198 bp deletion in *CCR5* (Table 3). Importantly, biallelic deletions were observed in 36.7% of embryos (Table 3).

**Table 3:** Genotype summary of *CCR5*-editing in CRISPR-Cas9 microinjected embryos.

Analysis	Total embryos	% Wild-type	% Heterozygote	% Homozygote	% CCR5 edited embryos
Whole embryo	60	46.7	16.7	36.7	53.3
Blastomeres	17	17.6	58.8	23.5	82.4

The *CCR5* gene region was evaluated in whole embryos and individual blastomeres of embryos. No signal was detected in 13 whole embryos and in individual blastomeres of one embryo, hence these embryos were excluded from the total embryos shown here.

Embryonic genome editing was also assessed in single blastomeres by PCR of 129 blastomeres from 18 developmentally arrested embryos (Table 4). Of note, all blastomeres were dissociated from arrested cleavage-stage embryos and analyzed for the *CCR5* PCR amplification product to assess genome editing events and the degree of mosaicism within an individual embryo. No PCR signal was detected in 43 blastomeres or any cells from one embryo. Analysis of individual blastomeres revealed diversity in genotypes at the *CCR5* locus within an individual embryo, where either biallelic *CCR5* deletions, a combination of wild-type or *CCR5* deleted alleles or all wild-type alleles were present (Table 4). Approximately 82% of the embryos assessed at the individual blastomere level contained *CCR5* deletions and 23.5% contained a biallelic deletion (Table 3). Of note, mosaicism was observed in 52.9% of the *CCR5*-edited embryos. The differences in editing efficiency (~53% of whole embryos and ~82% of embryos with individual blastomere analysis) may be attributed to fewer embryos being analyzed at the individual cell level (blastomeres from 18 embryos versus 73 whole embryos). Regardless of analysis method, biallelic embryonic genome editing was observed in ~ 23–37% of embryos.

**Table 4:** *CCR5* genotyping of individual blastomeres from CRISPR-Cas9 microinjected embryos.

	Total cells	Number of cells:				Genotype
		Homozygote	Heterozygote	Wild-type	No PCR signal	
Embryo1	8	0	6	1	1	Heterozygote, mosaic
Embryo2	8	0	4	0	4	Heterozygote
Embryo3	7	1	3	2	1	Heterozygote, mosaic
Embryo4	6	0	3	2	1	Heterozygote, mosaic
Embryo5	9	4	1	0	4	Heterozygote, mosaic
Embryo6	7	3	2	1	1	Heterozygote, mosaic
Embryo7	2	0	0	2	0	Wild-type
Embryo8	9	2	2	3	2	Heterozygote, mosaic
Embryo9	4	1	0	0	3	Homozygote
Embryo10	1	0	0	0	1	Unknown
Embryo11	4	1	0	0	3	Homozygote
Embryo12	4	2	0	0	2	Homozygote
Embryo13	5	0	0	3	2	Wild-type
Embryo14	Multiple*	1	0	0	0	Homozygote
Embryo15	13	4	3	1	5	Heterozygote, mosaic
Embryo16	14	3	3	2	6	Heterozygote, mosaic
Embryo17	16	0	0	13	3	Wild-type
Embryo18	12	5	1	2	5	Heterozygote, mosaic
Total	129	27	28	32	43	

Total cells represents the number of blastomeres dissociated from a developmentally arrested embryo, whereas the asterisk denotes a cluster of multiple blastomeres that could not be enzymatically or mechanically separated and here is only counted as 1.

### Selection of embryo recipients and embryo transfer

Circulating levels of progesterone, estradiol and in some cycles, luteinizing hormone (LH) were evaluated in potential embryo recipients from day 9–10 to 16 following the onset of menses. Ovarian cycles were analyzed for the decline in estradiol with a corresponding increase in progesterone indicative of ovulation. A total of 36 ovarian cycles were evaluated, where ovulation was observed in 14 cycles by a rise in progesterone and decline in estrogen (Supplementary Fig. S2A). The mean day of ovulation was 12.6 days post-onset of menses in 14 cycles analyzed. In five cycles where LH was assayed, the mean day of the LH peak was 12.6 days post-onset of menses (Supplementary Fig. S2B). In 22 cycles (~ 61% of cycles analyzed), ovulation was not observed, but rather the animal was either anovulatory (low progesterone, low estrogen; Supplementary Fig. S2C), in a persistent luteal phase with high progesterone (Supplementary Fig. S2D), or additional blood sampling outside the window of collection would have been needed for confirming the day of ovulation. Altogether, the evaluation of circulating hormone levels allows for identifying potential embryo recipients who have ovulated, although a large proportion of potential MCM embryo recipients were anovulatory or well into the luteal phase of their cycle.

Embryos were transferred to embryo recipients with confirmed and unconfirmed timing of ovulation. A total of 26 surgical oviductal and 13 non-surgical trans-cervical cannulations were attempted, in which embryos were successfully transferred to the oviduct or uterus in 20 and 12 cannulations, respectively (Supplementary Table S1). Pronuclear or 2-cell embryos were transferred to the oviduct and 4- to 16-cell embryos were transferred to the uterus. In three of each surgical and non-surgical cannulations, a confirmed ovulating female served as the embryo recipient. This is the first study in which cynomolgus macaque embryos were transferred at this research center, although rhesus macaque embryos have previously been successfully transferred using similar methodology^24^. Despite the transfer of 222 embryos (50 embryos to confirmed ovulating females), no pregnancies were obtained.

## Discussion

Human patients containing a homozygous 32 bp deletion in the *CCR5* gene have been shown to be resistant to HIV infection^1,2^, thus targeting of *CCR5* has received great attention for developing drug and gene therapies for HIV. NHP, such as MCMs, share similar immune responses and genetics with humans making them suitable models for HIV infection^12^. Mutating the *CCR5* locus in MCMs is particularly advantageous given the restricted MHC haplotypes of this sub-species^12,13^. Prior to this report, however, there were no studies specifically describing IVF or manipulation of MCM embryos, and others have observed poor development in in vitro fertilized embryos from MCMs (Carol Hanna and Jon Hennebold, Oregon National Primate Research Center, pers. comm.). In this study, we report for the first time in vitro development and CRISPR-Cas9 targeting of MCM embryos. By refining ovarian stimulation and in vitro culture conditions established for Chinese cynomolgus macaques, a cohort of mature MCM oocytes could be obtained and fertilized in vitro. Introduction of dual-guide sgRNAs targeting *CCR5* resulted in > 50% of the embryos containing mutations in *CCR5* with a third of these embryos containing biallelic mutations. While reduced embryonic development rates were observed in CRISPR-Cas9 microinjected embryos, the establishment of in vitro production methods and the observed high targeting rate offers promise for utilizing CRISPR-Cas9 technology to assess mutated gene function in MCMs.

In vitro embryo production methods have been established for cynomolgus macaques^21,22,25,26^, however, previous reports do not specify the origin of the cynomolgus macaques when using assisted reproductive technologies. For this study, we applied methods of ovarian stimulation and IVF established for domestic (i.e., Chinese origin CMs obtained from domestic breeding programs) to MCMs. An ovarian stimulation protocol similar to that used for rhesus^27^ or domestic cynomolgus macaques^21,22,28^ of twice daily FSH treatment for 8–10 days followed by laparoscopic retrieval between 26–32 h post-hCG treatment resulted in low recovery yields of mature oocytes. A larger proportion of mature oocytes were recovered upon extending the FSH treatment to 11–11.5 days and retrieving the oocytes at 38–40 h post-hCG treatment. This suggests that MCMs require a longer duration of stimulation for optimal in vivo oocyte maturation. The MCM oocytes were then fertilized using a similar protocol and culture medium as described by Curnow and Hayes^21^. Domestic cynomolgus macaque IVF rates range from ~ 49–69% for oocytes fertilized by ICSI with ~ 31–56% of those embryos developing to the blastocyst stage when cultured in vitro^22, 26, 28^. In this report, a cleavage rate of 72.7% and a blastocyst rate of 19.8% was achieved for control MCM embryos. Notably, the culture medium used in this study was reported to support a > 50% blastocyst rate in domestic cynomolgus macaques^21^. Hence, the reduced blastocyst rate in the present study suggests further optimization of culture conditions is needed to better support extended in vitro culture of MCM embryos.

Despite relatively low embryonic development rates, successful CRISPR-Cas9 targeting of *CCR5* was achieved in MCM embryos. Our group has previously demonstrated successful targeting of the *CCR5* gene using CRISPR-Cas9 technology in human iPSCs, which resulted in a 27% editing efficiency with ~ 41% of the edited cells containing homozygous or biallelic deletions in *CCR5*^20^. Applying a similar dual-guide approach to MCM embryos resulted in > 50% of the *CCR5* RNP microinjected embryos containing CCR5 deletions, and approximately a third of the CRISPR-Cas9 microinjected embryos containing biallelic deletions. This is one of few reports describing biallelic editing in NHP embryos. Wan et al.^29^ reported a 100% biallelic editing rate in 18 cynomolgus macaque embryos microinjected with CRISPR-Cas9 mRNAs targeting the *p53* gene. Similarly, Zhang et al.^30^ reported biallelic editing of *SIRT6* within 15 tissues of 3 cynomolgus macaque infants born from CRISPR-Cas9 targeted embryos. In these two reports a 100% biallelic editing rate was observed, however, the genes of interest were targeted using Cas9 mRNA and sgRNA rather than a Cas9-sgRNA RNP complex in one-cell embryos. In the current study, a *CCR5* RNP was selected for microinjection as it was suggested by Midic et al.^31^ that CRISPR-Cas9 targeting occurred more rapidly with RNP versus mRNA microinjections in rhesus macaque embryos. Regardless of the CRISPR-Cas9 construct, mosaic genome editing has been observed in studies using CRISPR-Cas9 genome editing approaches in NHP embryos^18,19,29,31,32^. A mosaic genome editing pattern was observed in the present study within ~ 50% of the *CCR5*-edited embryos. Notably, human patients with a naturally occurring, homozygous deletions in the *CCR5* gene show complete HIV resistance, whereas heterozygous individuals have delayed progression in HIV infection^1,6^. Hence, the introduction of either homozygous or heterozygous mutations in MCMs would be biologically relevant for disease modeling.

Decreased in vitro embryonic development and pregnancy rates have been observed in NHP embryos microinjected with higher concentrations and/or volumes of CRISPR-Cas9 mRNAs. Wan et al.^29^ showed that embryonic development was reduced by ~ 30% in cynomolgus macaque embryos microinjection with the highest concentration of 200 ng/μl Cas9 mRNA:10 ng/μl sgRNA compared to lower concentration of 100 ng/μl Cas9 mRNA:10 ng/μl sgRNA, while a 100% biallelic editing rate was observed for embryos microinjected with either concentration. Likewise, a study by Yao et al.^33^ reported that transfer of 27 cynomolgus macaque embryos microinjected with a volume of 4 pl of 100 ng/μl Cas9 and 50 ng/μl sgRNAs with 100 ng/μl donor plasmid produced no pregnancies following embryo transfer, whereas reducing the microinjection volume to 2 pl resulted in 5 pregnancies following the transfer of 42 embryos into 12 recipients. These studies collectively demonstrate a dose-dependent decrease in embryonic development upon microinjection with CRISPR-Cas9 constructs. In the present study, a *CCR5* RNP was used for CRISPR-Cas9 targeting in which a single concentration of 20 μM Cas9 protein was complexed with 50 mM of each sgRNA. Decreased cleavage and blastocyst rates were observed in Cas9 and *CCR5* RNP microinjected embryos compared to control. It is unclear whether reduced development is a result of the introduction of Cas9 or the microinjection process itself as sham injections were not performed in this study due to limited oocyte resources. Future studies are needed to further assess MCM embryonic development following sham microinjection and microinjection with differing concentrations and/or volumes of CRISPR-Cas9 constructs.

The failure of control and microinjected MCM embryos to implant following embryo transfer may be due to the lack of recipient cycle synchronicity, embryonic toxicity of the CRISPR-Cas9 constructs or embryonic lethality incurred by the genetic mutation. Upon monitoring candidate embryo transfer recipient cycles, ovulation was not observed in a large proportion of the cycles monitored. Hence, the lack of synchronicity did not allow for optimal timing for delivery of embryos to the oviduct or uterus. A larger cohort of embryo recipients with rigorous sampling of hormone levels may be needed in future studies to optimize the timing of embryo transfer in MCMs. There is a lack of knowledge regarding the regularity of MCM ovarian cycles, and thus, it is unclear whether the observations in this study are reflective of all MCMs or the WNPRC colony animals in the current study. Of note, blastocyst hatching was observed in control and Cas9 alone microinjected MCM embryos with long term in vitro culture in this study. The rate of live births following embryo transfer of genome-edited cynomolgus macaque embryos remains low. For example, Tu et al.^34^ reported that the transfer of 178 CRISPR-Cas9 microinjected embryos to 47 recipients resulted in 11 pregnancies and 6 live births. Notably, about 1.67–7.1% of the transferred CRISPR-Cas9 targeted cynomolgus macaque embryos result in a live edited offspring^16, 17, 29,30,32,33,35,36^. It is also possible that the CRISPR-Cas9-induced genetic mutations may contribute to embryonic lethality. A 39% reduction in live birth rate was observed following embryo transfer of *PKD-1* edited cynomolgus macaque embryos compared to wild-type embryos, whereas pregnancy rates were similar between groups^19^. The deletion in *CCR5* has been reported to occur at a frequency of < 1% in the human population^1,2^. Therefore, it is plausible that the *CCR5* gene has an unknown role in pre- or peri-implantation stages of embryonic development, and further study is needed to ascertain whether this rare mutation hinders embryonic survival.

In the present study, off-target effects were not evaluated as no off-target editing was observed in our cell based model^20^. Unpublished studies that have been deposited recently in the BioRxiv database (https://www.biorxiv.org/; Alanis-Lobato et al. 2020, Zuccaro et al. 2020) report large scale deletions on the same chromosome as the CRISPR-Cas9 target site within human embryos. Hence, it is conceivable that in embryos lacking PCR signal that the gene region was not able to be amplified because the PCR primer sites were within a large-scale deletion. While whole-genome DNA sequencing of *CCR5*-edited embryos was not performed in the present study, the recent suggestion that CRISPR-Cas9 may introduce large scale deletions warrants further analysis of the impact of gene targeting by CRISPR-Cas9 technology in NHP embryos.

## Conclusions

In conclusion, the present study describes the establishment of methods for MCM in vitro embryo culture and the successful targeting of the *CCR5* gene in MCM embryos. Monitoring MCM ovarian cycles with daily blood draws revealed a large proportion of cycles to be irregular and this warrants further exploration to assess if this is intrinsic to this sub-species. This finding may also underlie the problems faced by our research group and others in obtaining good quality oocytes and development of IVF embryos. Future studies are needed to further optimize MCM embryo culture conditions to better support blastocyst formation in vitro. Moreover, the concentration and volume of CRISPR-Cas9 RNP should be refined to achieve high editing efficiency without compromising embryonic development rates. The successful introduction of *CCR5* deletions in MCM embryos establishes a platform for futures studies to create a NHP model of SIV resistance in monkeys containing the *CCR5* deletion. Ultimately, the generation of an SIV resistance model will aid in our understanding of HIV disease progression and resistance as well as provide a system to further develop curative treatments and therapeutics.

## Methods

### Animals

Cynomolgus monkeys of (*Macaca fasciculata*) of Chinese (n = 5) and Mauritian (n = 31) origin were acquired from Alpha Genesis Inc (Yemassee, SC), Primate Products LLC (Miami, FL) (domestic CCMs) or Bioculture Ltd (Mauritius). A total of 32 females (5 Chinese and 27 Mauritian) were used in this study that were 4.3–12.1 years of age and weighed 3.28–6.7 kg. In addition, 4 MCM males served as semen donors that were 4.3–12.7 years of age and of a body weight ranging from 7.64 to 9.56 kg. All procedures were performed in accordance with the NIH Guide for the Care and Use of Laboratory Animals and under the approval of the University of Wisconsin College of Letters and Sciences and Vice Chancellor Office for Research and Graduate Education Institutional Animal Care and Use Committee.

### Ovarian stimulation and in vitro maturation of oocytes

Regularly cycling females underwent ovarian hyperstimulation beginning on days 1–4 of menses. Monkeys were administered 30 IU recombinant human follicle stimulating hormone (rhFSH) (IVF Prescription, Puregon) intramuscularly, twice-daily at twelve hour intervals for 11–11.5 days. An injection of 1000 IU recombinant human chorionic gonadotropin (hCG; IVF Prescription, Ovidrel) was administered in the evening on day 11 or 11.5. Laparoscopic oocyte aspirations were performed between 38–40 h post hCG injection in which the ovaries were manipulated to visualize and aspirate all visible follicles. Oocytes were aspirated into HTF-HEPES solution (Irvine Sci., cat no: 90126) supplemented with 3 mg/mL human albumin (MP Biomedicals, cat no: 823051), 0.28 mg/mL heparin (Sigma-Aldrich, cat no: H3149) and 0.28 mg/mL hyaluronidase (Sigma-Aldrich, cat no: H3884) and filtered through a 100 μM strainer (PluriSelect, cat no: 435010051) to remove blood clots and cumulus cells from oocytes. The oocytes were washed from the strainer and placed into maturation medium composed of CMRL 1066 medium (Thermo Fisher Scientific, cat no: 11530037) supplemented with 0.5 mM sodium pyruvate (Sigma-Aldrich, cat no: 2256), 2 mM Alanyl-glutamine (Sigma-Aldrich, cat no: G8541) and 20% fetal bovine serum (FBS; Peak Serum, cat no: PS-FB1, Wellington, CO, USA) as similarly described by Curnow and Hayes^21^.

### Semen collection and oocyte fertilization

Semen was collected from male MCMs by electroejaculation while under mild ketamine sedation. Following collection, semen was incubated at room temperature for 30 min to allow the coagulum to liquefy prior to processing. The coagulum was removed and sperm samples were washed twice in HEPES-TL (Caisson Laboratories, cat no: IVL01) supplemented with 0.1 mM sodium pyruvate and 3 mg/mL bovine serum albumin (Sigma-Aldrich, cat no: A8806), diluted as needed, and transferred into 7% polyvinylpyrrolidone (PVP) for ICSI.

Prior to fertilization, oocyte maturation was evaluated for progression to the metaphase II stage, denoted by the presence of an extruded polar body. The duration of oocyte maturation was approximately 4–5 h. Mature MII oocytes were fertilized by ICSI and transferred into Global medium. ICSI was performed without exposure to fluorescent light, since we noted its negative effect on MCM embryo development.

### CRISPR-Cas9 constructs and embryo microinjection

A *CCR5* deletion was introduced using two gRNAs designed to disrupt *CCR5* sequences within exon 2, including a 24-bp deletion region which was found to prevent functional *CCR5* expression in NHPs^23^. sgRNA#1 (GCUGCUGCCCAGUGGGACUU) and sgRNA#2 (GCUGUGUUUGCCUCUCUCCC) were synthesized by Synthego Corporation (Menlo Park, CA, USA). To generate the *CCR5* RNP complexes, 20 μM Cas9 protein containing two nuclear localization signals (2NLS) (PNA Bio, https://www.pnabio.com/products/CRISPR_Cas9.htm, Newbury Park, CA) and 50 mM of each sgRNA were dissolved in 10 mM Tris–HCl (pH 7.4) containing 0.1 mM EDTA and sterilized with a 0.2 mm filter. The mixture was kept at room temperature for 20–30 min and centrifuged for 1 min at 20,000×*g*. The supernatant (~ 3 μL) was then loaded into a microinjection needle and ~ 10–15 pl of the CRISPR-Cas9 RNP solution was injected into the oocyte cytoplasm between 5–7 h post-ICSI^37^. The microinjection pipette was calibrated prior to injection to ensure uniform delivery to the high viscosity of CRISPR/Cas9 solution. Calibration was performed by measurement of the size of the drop injected under mineral oil on plastic plate. Injected volume was verified after injection of 10 oocytes and corrected if needed.

### Embryo culture and time-lapse embryo imaging

Following fertilization, embryos were cultured in Global Total medium (Cooper Surgical, cat no: LGGT-030). Embryos were either cultured in cohorts in a standard incubator or cultured individually for time-lapse embryo imaging to assess developmental morphokinetics. MCM embryos were cultured in cohorts of 5–10 embryos per 40 μL drop of Global Total medium supplemented with 1 mg/mL human albumin (MP Biomedicals, cat no: 823051) under light mineral oil (Irvine Sci., Cat#9305) at 37 °C in l ow oxygen (5% CO_2_, 5% O_2_). Alternatively, embryos were cultured in a MIRI TL Time-Lapse incubator (Esco Medical, Denmark) to monitor embryo development. Individual embryos were placed into a microwell of a CultureCoin MIRI-TL dish (Esco Medical, Denmark) containing 25 μL of Global media overlaid with 3 mL of mineral oil. The medium was equilibrated for 3–4 h at 37 °C in low oxygen (5% CO_2_, 5% O_2_) prior to embryo culture. Individual microwells were then imaged every five minutes across five focal planes. The time of ICSI was determined as 0 h and the time of cleavage to 2-cells, 4-cells, and 8-cells and the initial timing of morula and blastocyst formation were annotated for each embryo.

### PCR analysis of whole embryos and single blastomeres

To isolate DNA from embryos, the zona pellucida was removed by treatment with 1 mg/mL of activated pronase E (Sigma-Aldrich, cat no: P2730) under mineral oil. The whole embryo was washed in calcium and magnesium free PBS with 0.2% EDTA and 1 mg/mL human albumin (MP Biomedicals, cat no: 823051). Single blastomeres could be obtained by gently pipetting the embryo. DNA was extracted and amplified from single blastomeres or whole embryos using a REPLI-G single cell kit (Qiagen, cat no: 150343). Quality control analysis of amplified DNA was performed using an Agilent Femto Pulse system (Agilent, Santa Clara, CA) to confirm a uniform yield of DNA product with the average product length of more than 9.4 kb. PCR was performed using primers that annealed to sequences upstream and downstream of the CCR5 target region: forward primer 612 (5′-TCAATGTGAAACAAATCGCAGC) and reverse primer 613 (5′-TCGTTTCGACACCGAAGCAG). The temperature profile was 98 °C for 2 min followed by 32 cycles of 98 °C for 30 s, 58 °C for 30 s, 72 °C for 40 s and a final extension step at 72 °C for 10 min. The PCR products were them run on a 1% agarose gel. The expected size of the unmodified (wild-type) *CCR5* PCR amplicon is 613 base pairs.

### Embryo recipient hormone analysis and embryo transfer

Blood samples were drawn from potential embryo recipients from day 9 to 16 post-onset of menses. Blood tubes were centrifuged at 1300 × *g* for 10 min at room temperature and isolated serum was stored at – 80 °C. Steroid hormones were extracted from a 400 μL aliquot of serum and evaluated for progesterone and estradiol levels using the protocol previously described with minor modifications to the LC method^38,39^. The inter-assay coefficient of variation (CV) was determined by pools of human and macaque serum and ranged from 6.09 to 14.65%. To evaluate luteinizing hormone (LH) levels, a radioimmunoassay (RIA) was performed as previously described^40^. Briefly, LH concentrations in serum samples were measured by RIA in duplicate using the recombinant cynomolgus LH kit from the Hormone and Peptide Program (Torrance, CA, USA). All samples were run in two assays; one assay was run using the rapid (2-day method) and another with the regular (3-day method). The sensitivity of the assay was 0.01 ng/tube. The intra-assay CV for the rapid assay was 2.21% and the regular was 3.76%.

Embryos were either transferred surgically by laparoscopic cannulation of the oviduct or non-surgically by trans-cervical cannulation using methods previously described for rhesus macaques^24^. Up to ten embryos were transferred to an individual recipient, where one to two-cell embryos were placed in the oviduct and 4- to 16-cell embryos were placed into the uterus.

### Statistical analysis

Statistical analysis was performed using either RStudio (https://rstudio.com/) or Graphpad Prism (https://www.graphpad.com/scientific-software/prism/) software. The statistical method used for each comparison is described in the table legend. Embryo development rates within MCM or CCM were compared using the fmsb package in RStudio to perform pairwise Fisher’s Exact tests with a post-hoc Bonferroni correction for multiple comparisons. For all other analyses, Graphpad Prism software was used.

### Disclaimer

The content is solely the responsibility of the authors and does not necessarily represent the official views of the National Institutes of Health.

## Supplementary information

Supplementary Information.
