# Supplementary material for: Genome editing of CCR5 by CRISPR-Cas9 in Mauritian cynomolgus macaque embryos
Source: Sci Rep. 2020 Oct 28;10:18457. doi: 10.1038/s41598-020-75295-z (PMC7595107; doi:10.1038/s41598-020-75295-z)
Supplement: Supplementary file 1 — Supplementary Information. [file 41598_2020_75295_MOESM1_ESM.pdf]

Genome editing of *CCR5* by CRISPR-Cas9 in mauritian cynomolgus macaque embryos

Keywords: *in vitro* fertilization (IVF), preimplantation embryo, genome editing, CCR5, CRISPR/Cas9

Jenna Kropp Schmidt[1†], Nick Strelchenko[1†], Mi Ae Park[1], Yun Hee Kim[1], Katherine D. Mean[1], Michele L. Schotzko[1], Hyun Jun Kang[1], Thaddeus G. Golos[1,2,3‡*], and Igor I. Slukvin[1,4,5‡*]

[1]Wisconsin National Primate Research Center, University of Wisconsin-Madison, Madison, WI, USA
[2]Department of Comparative Biosciences, University of Wisconsin-Madison, Madison, WI, USA
[3]Department of Obstetrics and Gynecology, University of Wisconsin-Madison, Madison, WI, USA
[4]Department of Pathology and Laboratory Medicine, University of Wisconsin-Madison, Madison, WI, USA
[5]Department of Cell and Regenerative Biology, University of Wisconsin-Madison, Madison, WI, USA

Grant Support: This research was funded by NIH grants R24OD021322 awarded to IIS and TGG, P51OD011106 awarded to the Wisconsin National Primate Research Center, University of Wisconsin-Madison, and K99 HD099154-01 awarded to JKS. This research was conducted at a facility constructed with support from Research Facilities Improvement Program grant numbers RR15459-01 and RR020141-01.

†Drs. Schmidt and Strelchenko equally contributed to this work.
‡Drs. Golos and Slukvin are co-senior authors.

***Correspondence:**
Dr. Thaddeus G. Golos, Department of Comparative Biosciences, Wisconsin National Primate Research Center, University of Wisconsin-Madison, 1220 Capitol Court, Madison, WI 53715; E-mail: golos@primate.wisc.edu

Dr. Igor I. Slukvin, Department of Pathology and Laboratory Medicine, Wisconsin National Primate Research Center, University of Wisconsin-Madison, 1220 Capitol Court, Madison, WI 53715; E-mail: islukvin@wisc.edu

Supplemental Figure S1. Full length gel electrophoresis images of data shown in Figure 4B and 4C. Gel electrophoresis images of whole embryos (A) and single blastomeres (B).

Supplemental Figure S2. Representative hormone profiles at differing stages within the ovarian cycle. Blood samples were collected between days 8-16 post the onset of menses and progesterone and estradiol-17beta levels were evaluated. In some cycles, LH levels were also assayed. A. LH confirmed ovulation on day 12 post-menses, B. ovulation on day 15 as determined by a decline in estrogen with a rise in progesterone, C. an anovulatory cycle, and D. an ovarian cycle within the luteal phase.

Supplemental Figure S1

A.

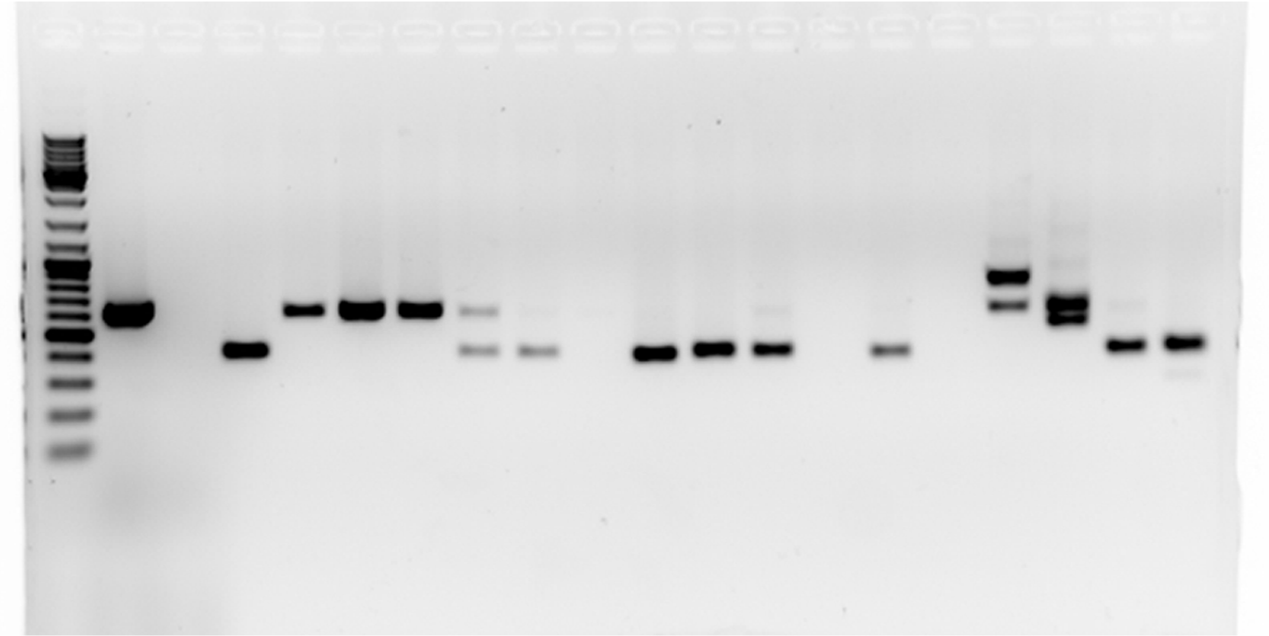

B.

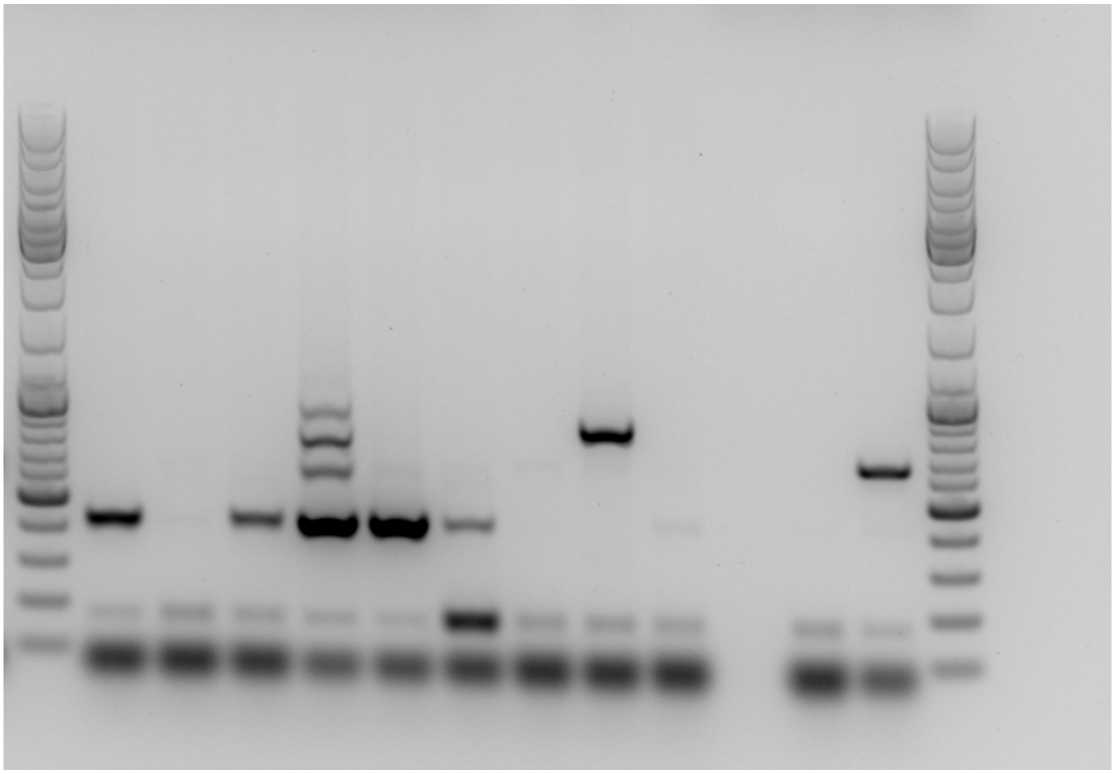

Supplemental Figure S2

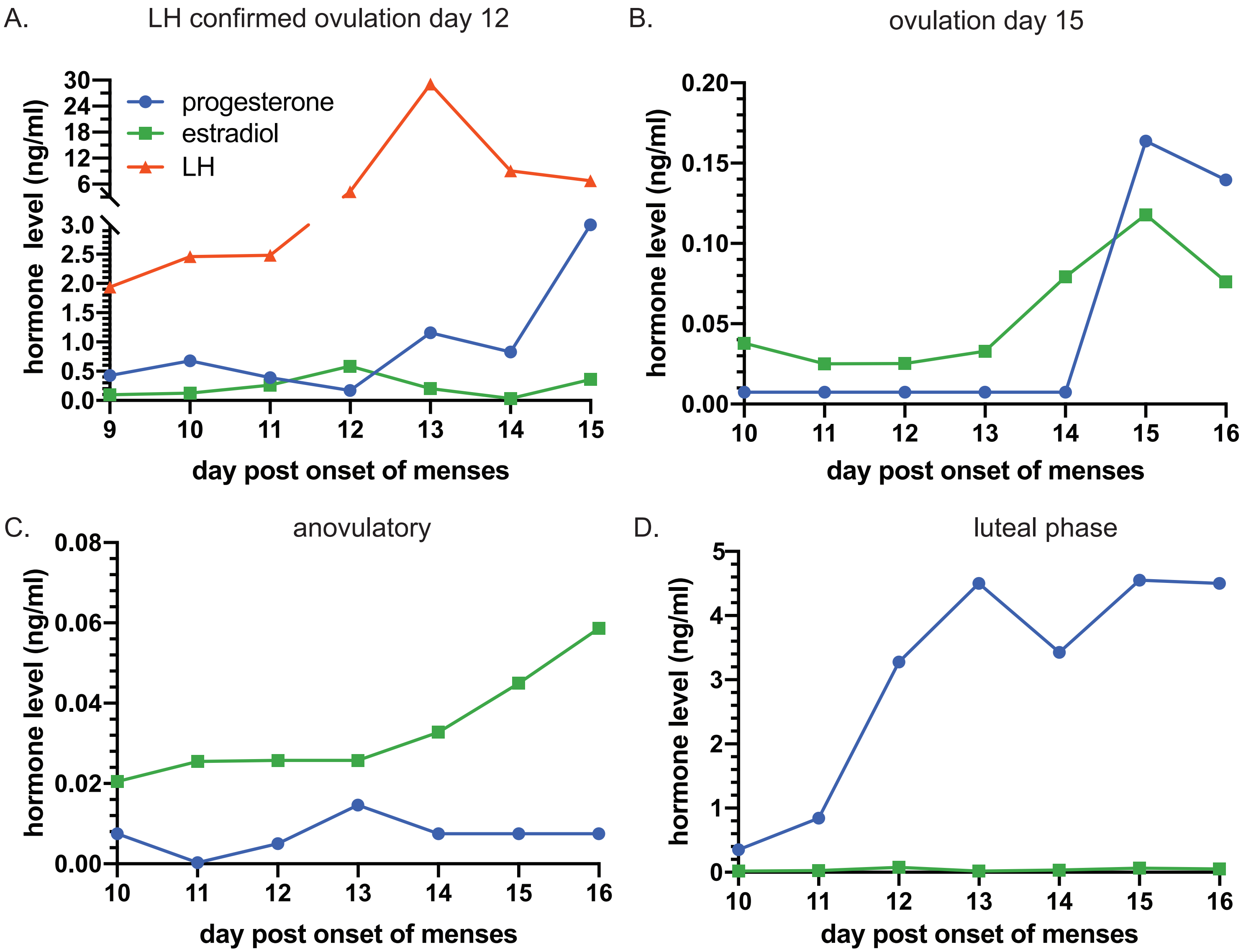

Supplemental Table S1. Summary of ovarian stimulation response in MCMs. A nonparametric Kruskal-wallis test with Dunn's correction for multiple comparisons was used for statistical analysis.

Stimulation	n	Total MII oocytes ± SD	Total oocytes ± SD
1	23	15.1 ± 18.3	30.0 ± 24.3
2	21	15.1 ± 14.2	22.5 ± 17.3
3	13	8.3 ± 11.9	16.3 ± 15.0
4	2	20.0 ± 22.6	31.0 ± 36.8
p-value		0.29	0.26

Supplemental Table S2. Summary of surgical and non-surgical embryo transfer attempts.

method	cannulation attempts	autologous transfers	successful cannulations	embryos transferred	transfers to confirmed cycle-matched recipients	embryos transferred to confirmed cycle-matched recipients
surgical	26	5	20	146	3	23
non-surgical	13	1	12	76	3	27
